# Receptor‐Triggered Peptide Self‐Assembly Enables Multivalent Recruitment of Endogenous Antibodies for Cancer Immunotherapy

**DOI:** 10.1002/advs.78074

**Published:** 2026-09-30

**Authors:** Yihui Wang, Xia Wu, Chenguang Zhao, Hong Han, Wensha Xie, Dan Yuan, Junfeng Shi

**Affiliations:** ^1^ Hunan Provincial Key Laboratory of Animal Models and Molecular Medicine State Key Laboratory of Chemo/Bio‐Sensing and Chemometrics School of Biomedical Sciences Hunan University Changsha Hunan China; ^2^ Department of Cardiology The Central Hospital of Xiangtan (The affiliated Hospital of Hunan University), Hunan University Xiangtan Hunan China; ^3^ Shenzhen Research Institute of Hunan University Shenzhen Guangdong China

**Keywords:** cancer immunotherapy, EGFR‐targeting peptide, endogenous antibodies, multivalent effect, self‐assembly

## Abstract

Monoclonal antibody–based immunotherapies have achieved remarkable clinical success but remain limited by variable patient responses, immune‐related toxicities, and the complexity of antibody production. Antibody‐recruiting molecules (ARMs) offer an alternative strategy by harnessing endogenous antibodies to induce immune‐mediated tumor clearance; however, their therapeutic efficacy is often constrained by insufficient antibody recruitment arising from weak hapten–antibody interactions. Here we report a receptor‐triggered supramolecular assembly strategy that converts monovalent antibody recruiters into multivalent immunotherapeutic platforms directly on cancer cell surfaces. An EGFR‐targeting peptide (GE11) was conjugated with a dinitrophenyl (DNP) hapten and a short self‐assembly motif to afford a monomeric construct that undergoes EGFR‐mediated in situ self‐assembly into fibrillar networks on EGFR‐overexpressing cells. This receptor‐guided supramolecular transformation generates a high‐density multivalent display of haptens on the cell membrane, enabling efficient recruitment of endogenous anti‐DNP antibodies. The resulting assemblies induce potent complement‐dependent cytotoxicity (CDC) against EGFR‐positive cancer cells in vitro and produce significant antitumor efficacy in tumor‐bearing mouse models with minimal systemic toxicity. This work establishes receptor‐mediated peptide self‐assembly as a general supramolecular strategy for transforming monovalent antibody recruiters into multivalent immunotherapeutic platforms, providing a new framework for the design of selective ARMs for cancer immunotherapy.

## Graphical abstract

Receptor‐triggered supramolecular self‐assembly converts monovalent antibody‐recruiting molecules into multivalent platforms directly on cancer cell membranes. The resulting fibrillar assemblies present clustered haptens that efficiently recruit endogenous antibodies, enabling selective complement‐mediated immune killing of EGFR‐positive cancer cells.

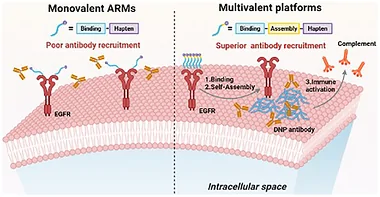

## 1 Introduction

Therapeutic monoclonal antibodies (mAbs) constitute a central pillar of modern cancer immunotherapy; however, their clinical utility is frequently constrained by immune‐related adverse events, limited patient response rates, and the high cost and complexity of protein production [1]. In addition, the generation of anti‐drug antibodies remains an unresolved immunogenicity challenge for antibody‐based therapeutics [2]. These limitations have stimulated the development of alternative strategy that harnesses endogenous antibodies to activate immune effector functions without the administration of exogenous proteins [3]. In this context, antibody‐recruiting molecules (ARMs) have emerged as a promising class of synthetic immune therapeutics, typically composed of an antibody‐binding domain (ABD) and a target‐binding domain (TBD), enabling the formation of ternary complexes between cancer cells and circulating antibodies [4]. To date, diverse tumor‐associated receptors, including folate receptor, integrin α_V_β_3_, PSMA, and uPAR, have been exploited as anchoring sites for ARMs [5, 6, 7]. Despite these advances, most reported ARMs rely on monovalent interactions at both the antibody‐ and target‐binding interfaces, resulting in inefficient antibody recruitment due to the intrinsically weak affinity between haptens and antibodies, as well as steric shielding by the tumor glycocalyx [8]. To enhance antibody recruitment, antibody‐recruiting polymers (ARPs) have been developed to present multivalent ABD and/or TBD motifs [9, 10, 11, 12]. These polymeric approaches significantly increase avidity but often lack precise control over spatial presentation and cellular selectivity. Moreover, nonspecific membrane anchoring and the use of synthetic polymer scaffolds raise concerns regarding off‐target effects and biocompatibility [13]. Consequently, a central challenge in ARM design is how to generate cell‐selective multivalent hapten displays from small‐molecule recruiters without relying on permanently multivalent synthetic scaffolds, which may compromise selectivity, pharmacokinetics, and translational feasibility.

Supramolecular self‐assembly offers a powerful chemical strategy for converting discrete molecular building blocks into higher‐order architectures through noncovalent interactions [14, 15]. Among these systems, peptide‐based self‐assembly is particularly attractive owing to its structural programmability, biodegradability, and intrinsic biocompatibility [16, 17, 18, 19, 20, 21]. Importantly, supramolecular assembly can transform weak monovalent interactions into strong multivalent binding through spatial clustering, a fundamental principle widely utilized in biological recognition processes [22]. Recent studies have shown that peptide self‐assembly on cell membranes can generate nanofibrous networks that actively modulate cellular behavior [23, 24, 25, 26, 27, 28]. In particular, tumor‐overexpressed receptors can further promote the localized accumulation and organization of monomeric precursors via ligand–receptor interactions, thereby triggering receptor‐mediated molecular assembly on the cell surface [29]. Such membrane‐confined assemblies create functional supramolecular interfaces directly on living cells and have been explored for regulating cell signaling, inhibiting metastasis, and enhancing drug retention [30, 31, 32, 33, 34, 35, 36, 37]. Despite these advances, the application of peptide self‐assembly to generate cell‐selective multivalent hapten displays for endogenous antibody recruitment in cancer immunotherapy remains largely unexplored.

To address the intrinsically weak affinity of small‐molecule haptens for endogenous antibodies, we report a receptor‐triggered supramolecular assembly strategy that enables multivalent hapten presentation through in situ molecular assembly on cancer cell membranes. Using a modular molecular design, we constructed monomeric peptide‐based antibody recruiters comprising an EGFR‐targeting peptide (GE11), a dinitrophenyl (DNP) hapten, and a short self‐assembly motif. A PEG linker was incorporated to optimize solubility and pharmacokinetic behavior. Upon binding to EGFR on cancer cell membranes, these monomers undergo receptor‐guided in situ supramolecular polymerization into fibrillar networks, thereby generating a spatially confined, high‐density multivalent hapten display directly on the cell surface (Figure 1A). The resulting supramolecular assemblies efficiently recruit anti‐DNP antibodies and induce complement‐dependent cytotoxicity (CDC) against EGFR‐positive cancer cells in vitro [38]. Furthermore, evaluation in tumor‐bearing mouse models demonstrates pronounced antitumor efficacy with minimal systemic toxicity. It is worth noting that Wang et al. recently developed a peptide self‐assembly antibody recruitment strategy (SARS) for tumor immunotherapy that is similar to ours [39]. By integrating receptor‐mediated peptide self‐assembly with hapten‐based antibody recruitment, this work establishes a supramolecular design framework for transforming monovalent recruiters into multivalent immunotherapeutic platforms, providing a new opportunity for cancer immunotherapy.

**FIGURE 1:**
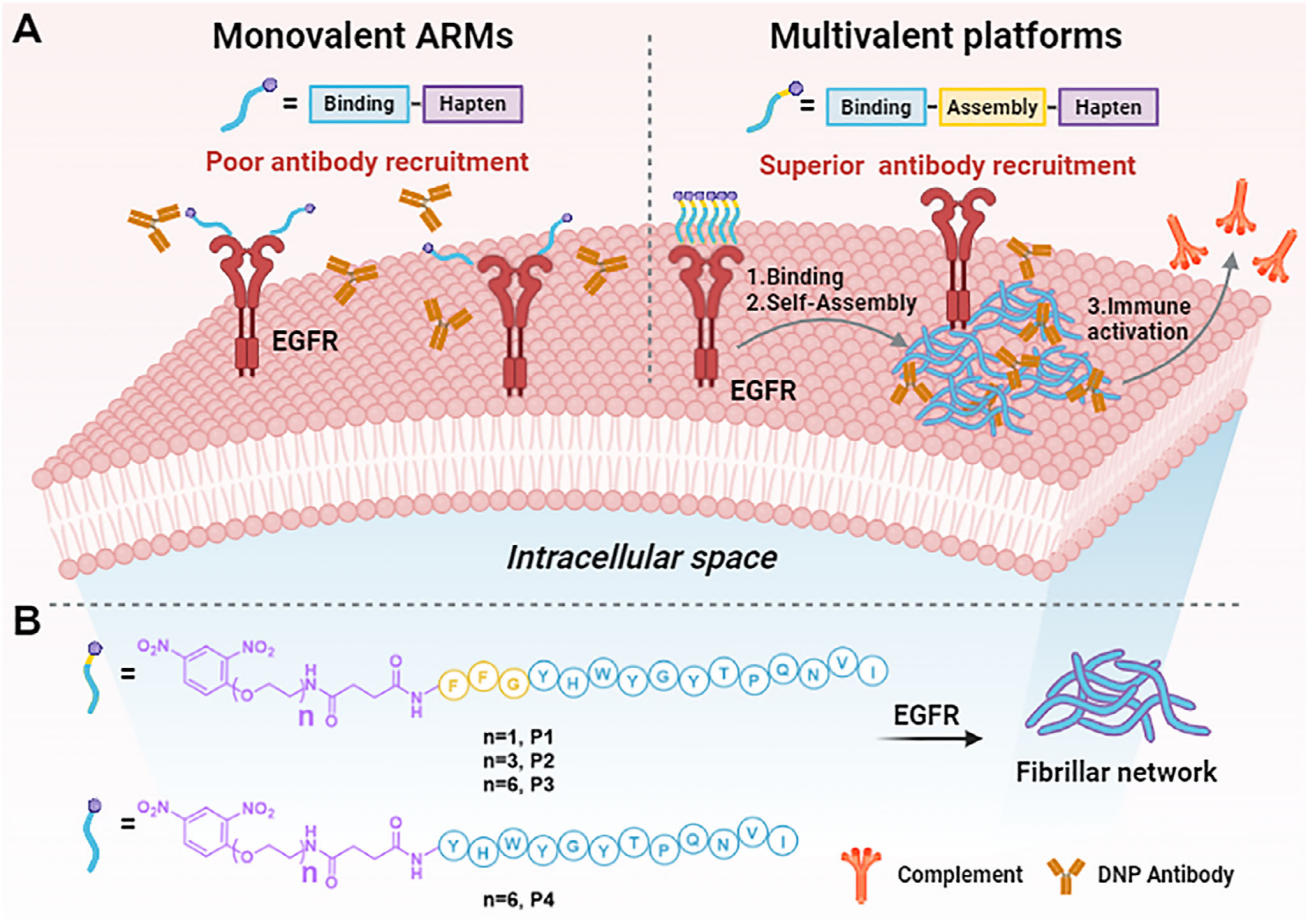
Concept of receptor‐triggered peptide self‐assembly for antibody recruitment. (A) Binding of the monomeric constructs to EGFR on the cancer cell membrane induces in situ supramolecular polymerization into fibrillar networks. The resulting multivalent display of DNP haptens efficiently recruits endogenous anti‐DNP antibodies to the cell surface, thereby labeling the cancer cell for elimination by innate immune effector mechanisms. (B) Chemical structures of the peptide–hapten conjugates and a schematic illustration of their receptor‐mediated supramolecular self‐assembly process.

## 2 Results and Discussion

### 2.1 Design and Synthesis of Peptide‐Hapten Conjugates

Based on the aforementioned rationale, a series of peptide‐based antibody recruiters (**P1**–**P4**) were designed to integrate three functional modules: an EGFR‐targeting peptide (GE11), a dinitrophenyl (DNP) hapten for antibody binding, and a self‐assembly motif. Diphenylalanine (FF) was selected as the supramolecular backbone owing to its well‐established propensity for β‐sheet‐driven assembly into ordered nanostructures, while **P4**, which lacks the FF motif, was designed as a non‐self‐assembling control molecule (Figure 1). The EGFR‐binding sequence YHWYGYTPQNVI (GE11) was introduced at the C‐terminus to confer receptor specificity, while glycine (G) and poly(ethylene glycol) (PEG) linkers of varying lengths were incorporated to modulate hydrophilicity and flexibility. To generate **P1**‐**P4**, GE11 peptide was conjugated with PEG‐modified DNP derivatives **S‐1**−**S‐3**, respectively (Figure 2). All compounds were fully characterized by liquid chromatography–mass spectrometry (LC–MS), high‐performance liquid chromatography (HPLC), and ^1^H Nuclear magnetic resonance (^1^H NMR), confirming their identity and purity (Figures S3–S21).

**FIGURE 2:**
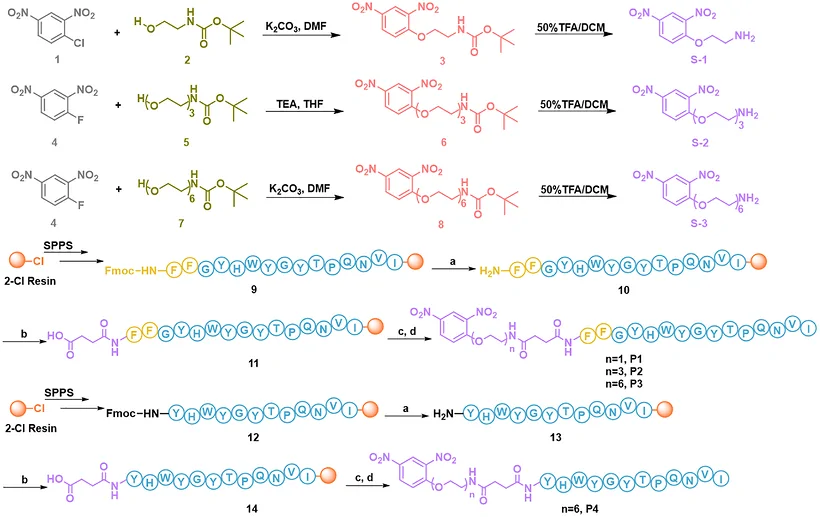
Synthetic route of GE11‐DNP Conjugates and their derivatives. Reagents and conditions: (a) 20% piperidine solution (dissolved in DMF), 20 min; (b) succinic anhydride, DIEA, DMF, r.t., 3 h; (c) compound **S‐1**/**S‐2**/**S‐3**, HCTU, DIEA, DMF, r.t., 10 h; (d) TFA/H2O/TIS (95:2.5:2.5), r.t., 3 h.

### 2.2 Receptor‐Triggered Self‐Assembly Enables Multivalent Antibody Recruitment

Given the EGFR‐targeting capability of GE11, the resulting GE11‐DNP conjugates were expected to selectively decorate EGFR‐positive tumor cells and recruit anti‐DNP antibodies. HeLa cells, which overexpress EGFR as verified by western blot analysis (Figure S22a), were therefore employed as a model system. Immunofluorescence imaging was first employed to evaluate antibody recruitment. As shown in Figure 3A, only punctate green fluorescence was detected on the membrane of cells treated with **P1** and **P2**, whereas intense and widespread green fluorescence was observed on the cell membrane after treatment with **P3,** further confirmed by 3D reconstructed imaging (Figure S23a,b). In contrast, negligible signal was observed in the control, **P4**‐treated, and GE11‐only groups. These results demonstrate that effective antibody recruitment requires the presence of targeting (GE11), antibody‐binding (DNP) motifs, and self‐assembly motifs. Moreover, the confocal images implied that antibody recruitment efficiency correlates with PEG linker length, identifying **P3**, which contains a PEG_6_ spacer, as providing the most favorable balance between hapten accessibility and receptor anchoring. Flow cytometry analysis further substantiated these observations, showing that treatment with 20 µm **P1**, **P2**, and **P3** resulted in progressively enhanced fluorescence intensities (Figure 3B).

**FIGURE 3:**
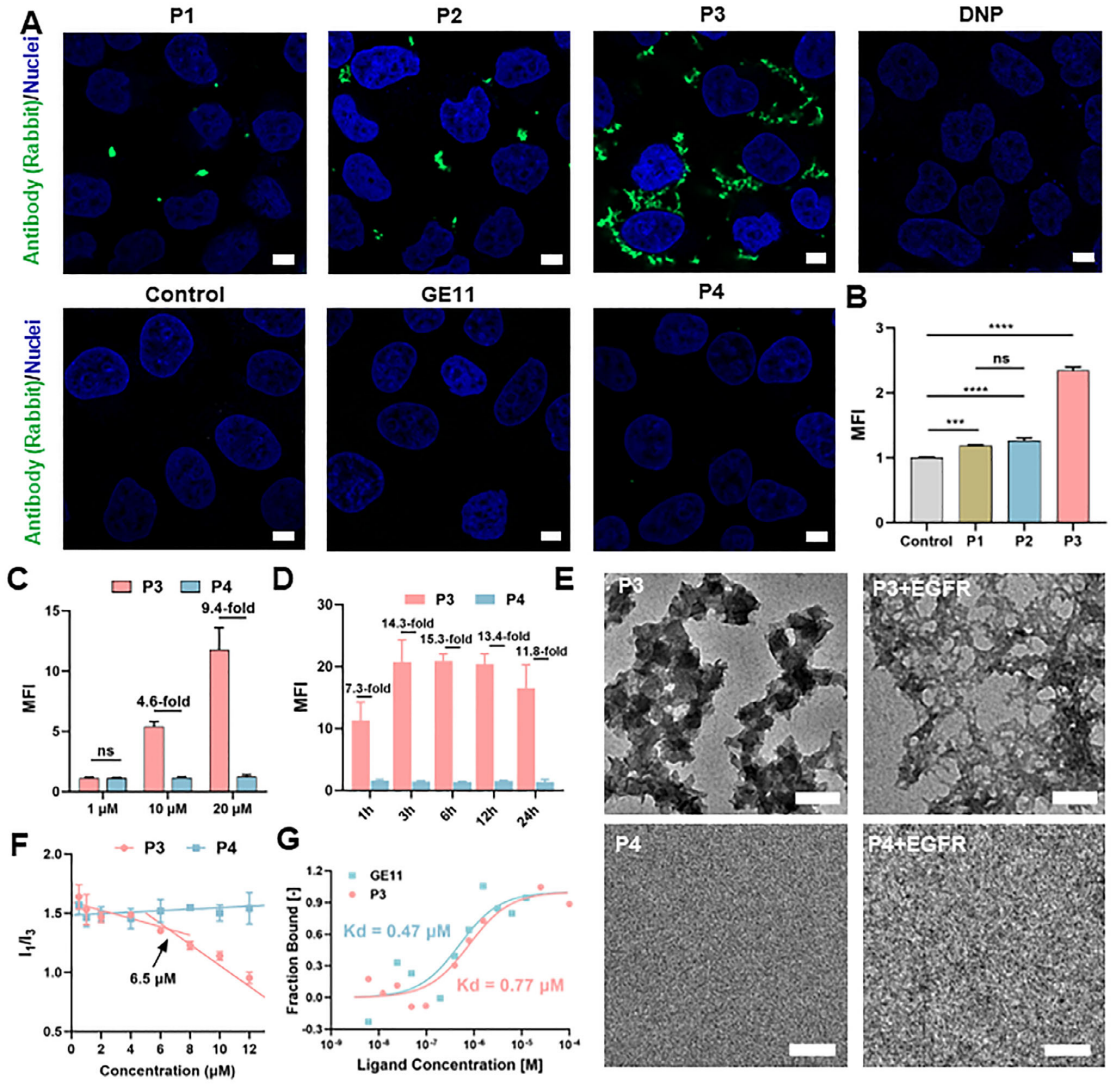
Antibody recruitment and supramolecular assembly of peptide–hapten conjugates on EGFR‐positive cells. (A) Immunofluorescence images of HeLa cells treated with 20 µM GE11, **P1**, **P2**, **P3**, **P4**, DNP, or PBS for 3 h. Scale bar: 5 µm. (B) Flow cytometry analysis of anti‐DNP antibody recruitment by **P1**, **P2**, and **P3** for 3 h. (C) Flow cytometry analysis of anti‐DNP antibody recruitment induced by **P3** and **P4** after 3 h incubation. (D) Flow cytometry analysis of anti‐DNP antibody recruitment by **P3** and **P4** at various time point. (E) TEM images of **P3** and **P4** incubated with EGFR protein or PBS for 24 h. Scale bar: 50 nm. (F) Determination of the CAC of **P3** by the ratio of pyrene fluorescence intensities at 373 nm (I_1_) and 384 nm (I_3_) as a function of peptide concentration. (G) The binding affinity between GE11/**P3** and EGFR was measured by MST assays.

Next, to directly evaluate the contribution of supramolecular self‐assembly on antibody recruitment. **P3**, which exhibits the strongest recruitment capability, and **P4**, which shares a similar sequence but lacks the diphenylalanine (FF) self‐assembly motif, were selected as comparative model systems. Quantitative flow cytometry analysis, consistent with this design principle, revealed that **P3** exhibited markedly enhanced, concentration‐ and time‐dependent antibody recruitment relative to **P4**. Specifically, at the concentration of 10 and 20 µm, **P3** yielded normalized mean fluorescence intensity (MFI) values of 5.4 and 11.8, respectively, after 3 h incubation, corresponding to 4.6‐ and 9.4‐fold enhancements over **P4**, respectively. Likewise, time‐dependent measurements at 20 µM revealed normalized MFI values of 11.3 at 1 h and 16.5 at 24 h, representing 7.3‐ and 11.8‐fold increases relative to **P4**. The fluorescence intensity associated with **P3** increased rapidly within the first 3 h and then reached a plateau, consistent with progressive accumulation and receptor‐guided self‐assembly of **P3** on the cell surface (Figure 3D). In addition to enhancing antibody recruitment, supramolecular assembly also improved molecular stability. Stability assays in 30% fetal bovine serum (FBS) showed that **P3** retained approximately 28.0% of its initial concentration after 8 h of incubation, whereas **P4** was completely degraded under identical conditions (Figure S27). Collectively, these results demonstrate that supramolecular self‐assembly not only amplifies antibody recruitment through multivalent hapten presentation, but also markedly enhances molecular stability and circulation persistence in biological media, thereby achieving superior therapeutic efficacy.

### 2.3 Supramolecular Assembly Enhances Antibody Recruitment Efficiency

To further elucidate the role of supramolecular assembly in antibody recruitment, we investigated the assembly behavior of **P3** and **P4** by transmission electron microscopy (TEM). **P3** alone predominantly formed amorphous aggregates, whereas incubation of **P3** (10 µM) with EGFR (25 nM) induced the formation of extended fibrillar networks (Figure 3E). In contrast, no ordered assemblies were observed for **P4** under identical conditions. These results indicate that the self‐assembly motif is essential for supramolecular polymerization and further suggest that EGFR may serve as a nucleating or organizing scaffold that promotes receptor‐triggered in situ assembly of **P3** into fibrillar architectures on the cell membrane [40, 41]. As shown in Figure S25, **P3** underwent receptor‐triggered morphological transformation into extended fibrillar networks in BTP buffer (pH 7.4), PBS (pH 6.5), and PBS (pH 8.5). These results indicate that the supramolecular assembly of **P3** is tolerant to the tested buffer and pH variations, supporting the robustness of receptor‐triggered assembly under physiologically relevant conditions. The critical aggregation concentration (CAC) of **P3** was determined to be 6.5 µm (Figure 3F). We next examined whether supramolecular modification affected the EGFR‐binding capability of **P3**. Microscale thermophoresis (MST) measurements showed that **P3** retained a high affinity for EGFR (Kd = 0.77 µm), comparable to that of the parental ligand GE11 (Kd = 0.47 µM). Collectively, these findings establish that receptor‐triggered self‐assembly is a critical determinant of antibody recruitment efficiency. The conversion of monomeric **P3** into supramolecular fibrillar networks generates a high‐density, multivalent display of DNP haptens directly on the cancer cell surface, thereby markedly enhancing anti‐DNP antibody binding through avidity effects.

To further evaluate the recruitment capacity and selectivity of **P3** toward anti‐DNP antibodies, we performed immunofluorescence studies using both human and mouse anti‐DNP antibodies. As shown in Figure 4A, prominent fluorescent signals were observed on the cell membranes of EGFR‐positive cell lines, including HeLa, MDA‐MB‐231, and A549, whereas negligible signals were detected in EGFR‐negative NHDF cells. These results demonstrate that antibody recruitment by **P3** is dependent on the presence of the target receptor. We next compared the antibody‐recruiting capability of **P3** and **P4** in tumor tissue. Subcutaneous HeLa tumors were established in BALB/c *nu/nu* mice, excised, and sectioned. The tissue sections were treated with **P3** or **P4**, followed by incubation with anti‐DNP antibodies and AF488‐labeled secondary antibodies, according to the experimental workflow shown in Figure 4B. Confocal microscopy revealed substantially stronger anti‐DNP antibody recruitment in **P3**‐treated tumor sections than in **P4**‐treated sections (Figure 4C). In particular, **P3** induced pronounced antibody accumulation at tumor cell membranes, together with signals associated with higher‐order supramolecular structures, whereas **P4** showed minimal antibody recruitment. These observations are consistent with the flow cytometry results obtained in vitro, which demonstrated markedly enhanced antibody recruitment by **P3** compared with **P4**. On the basis of its receptor‐triggered assembly, preserved EGFR‐binding capability, and superior antibody‐recruiting activity, **P3** was selected for subsequent mechanistic and therapeutic studies.

**FIGURE 4:**
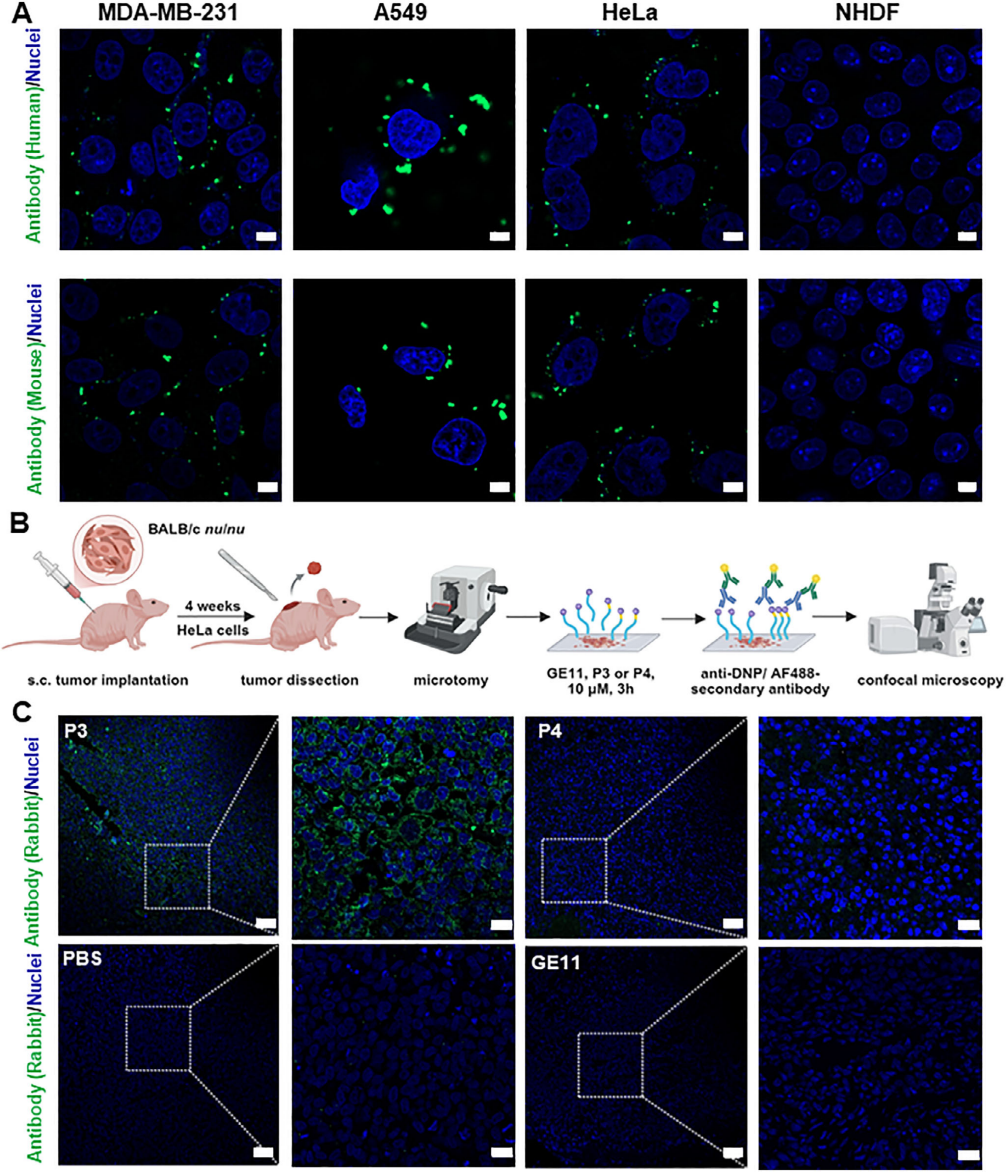
Supramolecular assembly of P3 enhances antibody recruitment in EGFR‐positive cells and tumor tissues. (A) Immunofluorescence images of NHDF, HeLa, MDA‐MB‐231, and A549 cells treated with 10 µm P3 for 3 h, followed by incubation with human or mouse anti‐DNP antibodies (20 µg/mL). Scale bar: 5 µm. (B) Schematic illustration of the experimental workflow for evaluating **P3**‐, **P4**‐, and GE11‐mediated antibody recruitment in HeLa tumor tissues. (C) Confocal microscopy images of HeLa tumor tissue sections treated with **P3**, **P4**, or GE11, followed by incubation with anti‐DNP antibodies and AF488‐labeled secondary antibodies. Scale bars: 50 µm for large‐field images and 20 µm for magnified images.

### 2.4 Receptor‐Mediated Assembly Governs Selective Cell Surface Localization

Next, we examined whether the GE11–DNP conjugates could selectively recruit anti‐DNP antibodies to EGFR‐positive cells, thereby validating the receptor‐mediated recruitment mechanism. As expected, pretreatment or co‐incubation with free GE11 as a competitive EGFR ligand markedly attenuated fluorescence signals on HeLa cell membranes (Figure 5A). A corresponding decrease in normalized MFI was also observed by flow cytometry (Figure 5C), confirming the EGFR dependence of antibody recruitment by **P3**. We further evaluated the cellular selectivity of **P3** by comparing its antibody‐recruiting activity in EGFR‐positive HeLa, MDA‐MB‐231, and A549 cells with that in EGFR‐negative normal human dermal fibroblasts (NHDF; Figure S22). Confocal fluorescence imaging revealed strong membrane‐associated fluorescence in all three EGFR‐positive cancer cell lines following **P3** treatment, whereas only weak fluorescence was detected in NHDF cells under identical conditions (Figure 5A,B). These observations were corroborated by flow cytometry, which showed that the normalized MFI of HeLa cells reached 6.5, corresponding to a 2.3‐fold increase relative to NHDF cells (Figure 5D). Collectively, these results demonstrate that **P3** exhibits high selectivity toward cells with elevated EGFR expression.

**FIGURE 5:**
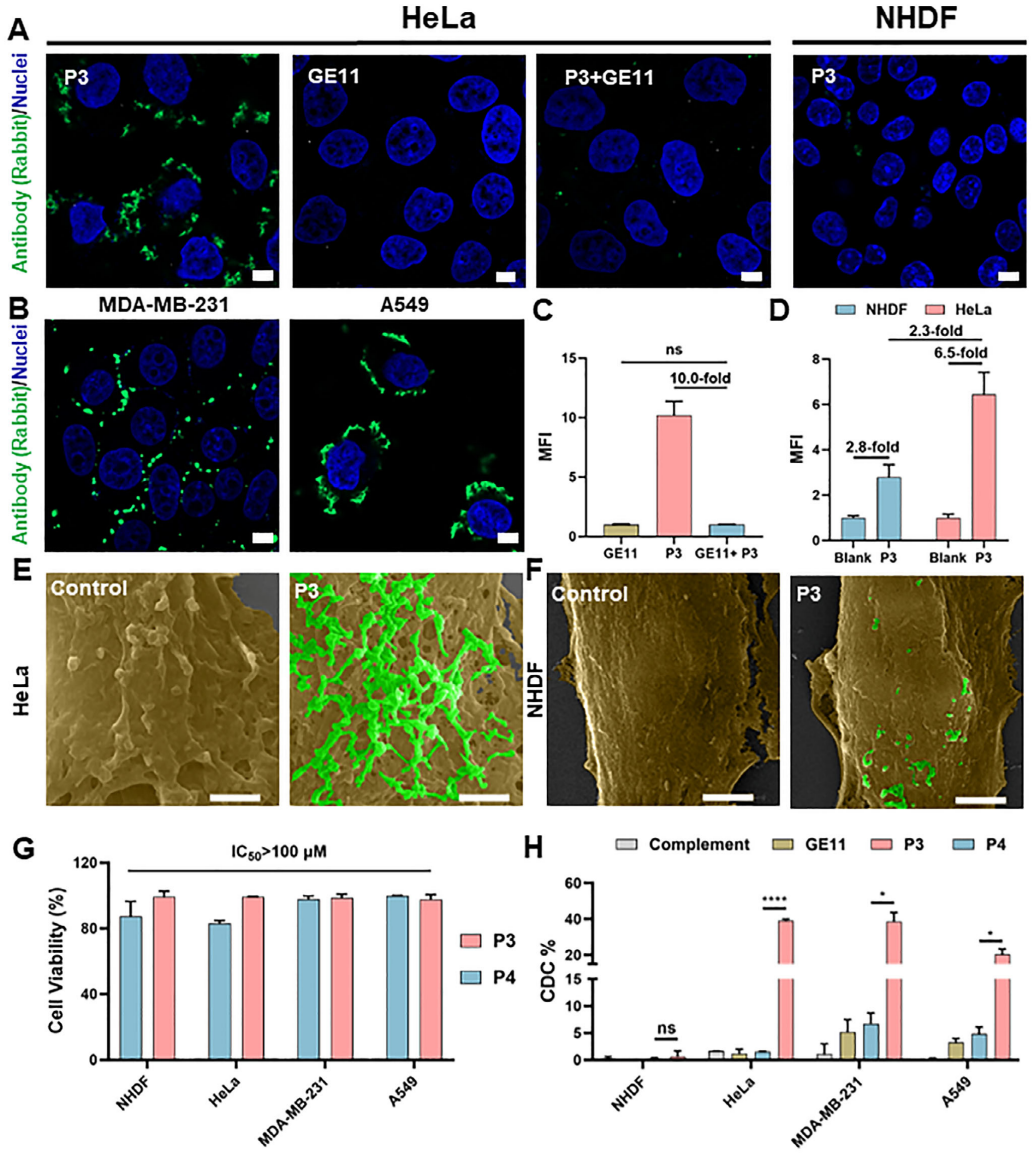
EGFR‐dependent selectivity, supramolecular assembly, and immune activation of P3. (A) Immunofluorescence images of HeLa cells incubated with 10 µm GE11, **P3**, and GE11+**P3** (EGFR competition) for 3 h. Scale bar: 5 µm. (B) Immunofluorescence images of MDA‐MB‐231 and A549 cells incubated with 10 µm **P3** for 3 h. Scale bar: 5 µm. (C) Flow cytometry analysis of anti‐DNP antibody recruitment induced with10 µM GE11, **P3**, and GE11+**P3** on HeLa cells for 3 h. (D) Flow cytometry analysis of anti‐DNP antibody recruitment induced with 10 µm **P3** on HeLa and NHDF cells for 3 h. (E,F) Pseudocolor scanning electron microscopy (SEM) images showing fibrillar networks of **P3** (green) assembled on the membranes of HeLa cells (E) and NHDF cells (F) (yellow) after incubation with 100 µm **P3** for 24 h. Scale bar: 2 µm. (G) Cell viability of NHDF, HeLa, MDA‐MB‐231, and A549 cells after 48 h incubation with 100 µm **P3** or **P4**, as determined by a CCK‐8 assay. (H) Complement‐dependent cytotoxicity (CDC) induced by 10 µm GE11, **P3**, or **P4** in NHDF, HeLa, MDA‐MB‐231, and A549 cells after 3 h incubation, followed by treatment with 10% rabbit complement (RC) for 10 h.

To directly visualize the selective self‐assembly of **P3** on cell membranes, scanning electron microscopy (SEM) was employed to examine membrane surface morphologies. Untreated HeLa and NHDF cells displayed smooth membrane surface (Figure 5E,F; Figure S26a,b). After incubation with **P3** for 3 h, extensive fibrillar network structures were clearly observed on the surfaces of HeLa cells (Figure 5E; Figure S26a). In contrast, no such assemblies were detected on NHDF cells treated with **P3** (Figure 5F; Figure S26b). These observations indicate that **P3** selectively binds to EGFR‐overexpressing tumor cells and undergoes receptor‐triggered self‐assembly into fibrillar networks on the cell membrane.

### 2.5 Supramolecular Assembly Induces Selective Immune Activation via CDC

Having established the selective recruitment of anti‐DNP antibodies to EGFR‐positive cells, we next investigated whether membrane‐bound antibodies could activate complement‐dependent cytotoxicity (CDC). For CDC assays, cells were treated with PBS, 10 µm GE11, **P3**, or **P4** in the presence of anti‐DNP antibodies and 10% rabbit complement (RC), followed by quantification of cell lysis using a CCK‐8 assay. As shown in Figure 5D,G and Figure S27, HeLa MDA‐MB‐231 and A549 cells treated with **P3** exhibited a cell lysis at range of 20.4%–39.2%, consistent with its superior antibody recruitment capability among all tested compounds. In contrast, NHDF cells (EGFR‐negative) showed minimal lysis (0.5%), comparable to those observed for PBS and **P4** controls. These results demonstrate that hapten functionalization confers immune effector activity on the targeting peptide GE11 by enabling endogenous antibody recruitment, and that CDC‐mediated cytotoxicity induced by **P3** is highly selective for EGFR‐positive tumor cells. Importantly, **P3** exhibited no detectable cytotoxicity toward primary human blood cells at concentrations up to 100 µM after 24 h of incubation (Figure S28). Taken together, these findings suggest that, in vivo, **P3** is expected to achieve enhanced antitumor efficacy through the receptor targeting, supramolecular self‐assembly, and multivalent antibody‐mediated immune activation, surpassing the therapeutic potential of GE11 alone.

### 2.6 In Vivo Antitumor Efficacy and Biosafety Evaluation

To evaluate the antitumor efficacy of the GE11‐DNP conjugates, BALB/c mice were immunized with OVA‐DNP to obtain the anti‐DNP serum (Figures S29 and S30), and BALB/c nude mice were subcutaneously implanted with HeLa. HeLa subcutaneous xenograft models were treated every two days with **P3** or **P4** at a dose of 10 mg kg^−^^1^. After two weeks of treatment, tumors were excised for photographic documentation, weighing, and quantitative analysis (Figure 6A). **P3** demonstrated marked superior antitumor activity compared to **P4** (Figure 6B–D). Mice treated with **P3** showed a pronounced suppression of tumor growth, with a mean tumor volume of 264.1 mm^3^, versus 1264.3 mm^3^ for PBS‐treated and 1003.0 mm^3^ for **P4**‐treated mice. Accordingly, the tumor growth inhibition (TGI) rate of **P3** reached 79.1%, substantially higher than that of **P4** (20.7%). These results demonstrate that incorporation of a self‐assembly motif enhances the in vivo antitumor efficacy of GE11–DNP conjugates in the presence of anti‐DNP antibodies.

**FIGURE 6:**
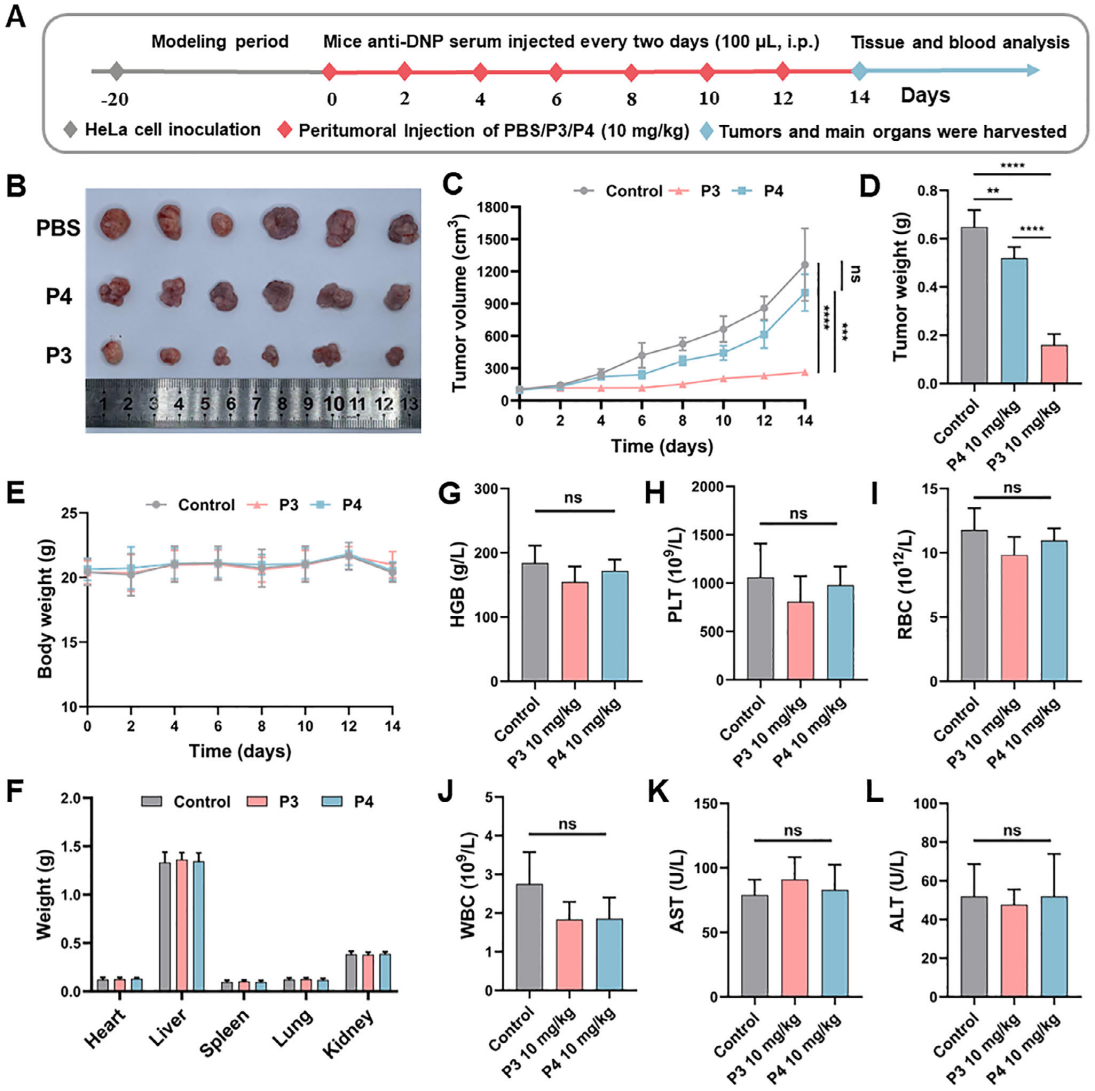
In vivo antitumor efficacy and biosafety evaluation. (A) Schematic illustration of the treatment regimen for subcutaneous HeLa tumor–bearing mice receiving PBS, **P3** (10 mg kg^−^^1^), or **P4** (10 mg kg^−^^1^) by peritumoral injection every two days. (B) Representative photographs of excised HeLa tumors on day 14. (C) Tumor growth curves of HeLa xenograft–bearing mice subjected to indicated treatments over 14 days (n = 6 biologically independent mice). (D) Tumor weights of the different treatment groups on day 14. (E) Body weight changes of mice in each treatment group during the 14‐day treatment period. (F–L) Organ weights and systemic toxicity indicators, including hematological parameters (HGB, PLT, RBC, and WBC) and liver function markers (AST and ALT), measured after sacrifice on day 14. (n = 6).

The systemic safety profile of **P3** was subsequently assessed. No significant differences in body weight were observed among the treatment groups throughout the study (Figure 6E), and the organ weights of mice treated with **P3** were comparable to those of the PBS group (Figure 6F). Serum biochemical markers of hepatotoxicity (AST and ALT) and hematological parameters (HGB, PLT, RBC, and WBC) remained within normal ranges (Figure 6G–L). Histological examination of major organs revealed no evidence of inflammatory infiltration or necrotic lesions (Figure S31), confirming the absence of overt systemic toxicity. Examination of tumor tissues further supported the therapeutic efficacy of **P3**. Hematoxylin and eosin (H&E) staining showed densely packed tumor cells with intact nuclear morphology in the PBS and **P4** groups, consistent with continued tumor growth, whereas tumors from **P3**‐treated mice displayed extensive necrotic regions with sparse cells and nuclear condensation (Figure S32). Collectively, these results establish **P3** as an effective peptidomimetic immunotherapeutic that selectively targets EGFR‐positive tumors while maintaining a favorable in vivo safety profile.

## 3 Conclusion

In summary, we have established a receptor‐triggered supramolecular self‐assembly strategy that enables highly efficient endogenous antibody recruitment on EGFR‐positive cancer cell membranes. A monomeric GE11‐DNP conjugate incorporating a short self‐assembly motif undergoes EGFR‐mediated in situ polymerization into fibrillar networks, thereby generating a spatially confined, multivalent hapten display. This supramolecular transformation substantially enhances antibody recruitment relative to non‐assembling analogues and leads to robust complement‐dependent cytotoxicity against EGFR‐overexpressing cancer cells in vitro, as well as pronounced antitumor efficacy in vivo. Beyond its immediate therapeutic impact, this work introduces a general chemical principle for transforming monovalent hapten‐based recruiters into multivalent immunotherapeutic platforms through receptor‐guided self‐assembly. Notably, the intrinsic aggregation tendency of **P3** in the absence of EGFR binding represents an important consideration for its further in vivo development. This limitation could potentially be addressed through molecular engineering of the self‐assembly motif and incorporation of additional hydrophilic functionalities. For example, hydrophilic or stimulus‐responsive motifs, including phosphatase‐responsive phosphate groups or glucose‐derived units, could be introduced to improve aqueous solubility and suppress nonspecific aggregation while retaining receptor‐triggered assembly [42, 43]. Such molecular optimization may further improve the balance between systemic stability, tumor accumulation, and target‐cell‐selective supramolecular assembly. The modularity of this molecular design permits straightforward adaptation to alternative targeting ligands and hapten motifs, offering a versatile and tunable framework for the development of next‐generation antibody‐recruiting systems.

## 4 Experimental Section

### 4.1 Materials

Fmoc‐protected amino acid, 2‐chlorotrityl chloride resin, and 2‐(1H‐benzotriazol‐1‐yl)‐1,1,3,3‐tetramethyluronium hexafluorophosphate (HBTU) were obtained from GL Biochem (Shanghai, China). N,N‐diisopropylethylamine (DIPEA), 2,2,2‐trifluoroethanol (TFE), trifluoroacetic acid (TFA), and triisopropylsilane were purchased from Energy Chemical (Shanghai, China). Methyl thiazolyl tetrazolium Cell Counting Kit‐8 (CCK‐8) was sourced from APExBIO (Houston, TX). DMEM was acquired from Gibco (Shanghai, China), and fetal bovine serum (FBS) was purchased from Adamas‐life (Shanghai, China). Hoechst and OVA were obtained from Thermo Fisher Scientific Inc. (Waltham, MA). Anti‐DNP antibody was procured from Seebio Biotech Co., Ltd. (Shanghai, China). Rabbit complement was supplied by Pel‐Freez Biologicals (Rogers, AR). Alexa Fluor 488‐labeled goat anti‐rabbit antibody (H+L), anti‐β‐actin, and anti‐EGFR antibody were purchased from Proteintech Group, Inc. (Wuhan, China). All other reagents were obtained from commercial sources if not otherwise indicated.

### 4.2 Cell Culture

HeLa and NHDF cell lines were cultured in DMEM medium (Gibco) supplemented with 10% FBS (Tansoole) and 1% penicillin‐streptomycin (Adamas Life). Both cell lines were cultured at 37°C in a humidified incubator with a 5% CO_2_ atmosphere.

### 4.3 Antibody Recruiting Capacity Assay

*Immunofluorescence Assay*. HeLa or NHDF cells were seeded in confocal dishes and cultured overnight in DMEM supplemented with 10% FBS. Following this incubation, the cells were treated with PBS, **P1**, **P2**, **P3**, **P4**, or GE11 at 37°C for 3 h. After treatment, the cells were washed with PBS and fixed with 4% formaldehyde for 15 min. They were blocked with 2% BSA in PBS for 1 h. Subsequent to blocking and PBS washing, the cells were incubated with rabbit anti‐DNP antibody (20 µg/mL, in 1% BSA) for 15 min, followed by a 1 h incubation in the dark with Alexa Fluor 488‐labeled goat anti‐rabbit antibody (H+L) at a 1:1000 dilution. Nuclei were stained with Hoechst for 15 min, and the cells were washed three times with PBS. Finally, 1 mL of PBS was added to the dishes, which were immediately transferred to a microscope stage for imaging. Cell imaging was initiated immediately, with the imaging parameters being kept constant throughout the experiment.

*Flow Cytometry Assay*. After incubation with PBS, **P1**, **P2**, **P3**, **P4**, or GE11 at 37°C for 3 h, the cells were harvested and fixed with 4% formaldehyde for 15 min. Fixed cells were then blocked with 2% BSA in PBS for 1 h. Following the PBS wash, the cells were incubated for 1 h with rabbit anti‐DNP antibody (20 µg/mL, in 1% BSA), and subsequently for 1 h in the dark with Alexa Fluor 488‐labeled goat anti‐rabbit antibody (H+L) at a 1:1000 dilution. After a final wash, the cell suspensions were filtered through a 200‐mesh sieve and analyzed by flow cytometry.

### 4.4 Critical Aggregation Concentration (CAC) Measurements

In a typical experiment, a series of solution **P3** or **P4** at different concentrations were prepared in PBS buffer. After incubating with Pyrene (20 µm) in 37°C for 24 h, the fluorescence spectra were recorded between 360 and 450 nm (λex = 334 nm) to determine the CAC.

### 4.5 Transmission Electron Microscopy (TEM) Imaging

Stock solutions of **P3** and **P4** were prepared in PBS at concentrations of 20 µm, while epidermal growth factor receptor (EGFR) was diluted to 50 nm in PBS. Four distinct sample mixtures were subsequently formulated and incubated for a total of 24 h within 37°C: (1) 10 µL of 20 µm **P3** combined with 10 µL PBS, (2) 10 µL of 20 µm **P3** mixed with 10 µL of 50 nM EGFR, (3) 10 µL of 20 µm **P4** added to 10 µL PBS, and (4) 10 µL of 20 µm **P4** blended with 10 µL of 50 nm EGFR. All solutions were equilibrated to room temperature prior to further processing. 10 µL solution sample was dropped onto super thin carbon‐coated copper grids (400 meshes) and allowed to sit for 1 min at room temperature. Then, excess sample solution was removed by gently blotting the edge of the grid with filter paper. Next, the grid was washed with 10 µL D.I. water for 1 min at room temperature. The extra D.I. water was removed from the edge of grid with filter paper (repeat this step 3 times). Finally, 10 µL uranyl acetate (1% v/v) was dropped onto the sample side of the grid and left for 1 min. The extra uranyl acetate was removed from the edge of grids with filter paper (repeat this step 3 times). The grid was then air‐dried before imaging. Images were taken with a JEOL JEM‐2100Plus transmission electron microscope.

### 4.6 Stability Experiment

In a typical experiment, 50 µL of 1 wt% solution of **P3** and **P4** was treated with 200 µL 30% FBS at 37°C with shaking of 50 rpm. The sample was taken out at the desired time and mixed with 110 µL of acetonitrile. The samples were then analyzed and quantified by analytical HPLC. Each time point was performed in triplicate.

### 4.7 Scanning Electron Microscopy (SEM) Imaging

HeLa and NHDF cells were treated with 100 µM **P3** for 24 h. After incubation, the cells were washed three times with PBS and fixed with 4.0% formaldehyde in PBS for 10 min at room temperature. Following three additional PBS washes, the samples were dehydrated through a graded ethanol series (70, 75, 80, 85, 90%, 95%, and 100% in deionized water) and then placed in 100% tertiary butanol. Finally, the dehydrated samples were lyophilized, and the resulting powder was mounted on copper tape, sputter‐coated with gold, and observed using a scanning electron microscope (Hitachi S‐4800).

### 4.8 Western Blotting Assay

HeLa and NHDF cells were seeded in six‐well plate and cultured for 24 h. After incubation, the cells were lysed in RIPA lysis buffer and boiled in sodium dodecyl sulfate sample (SDS) loading buffer. The samples were separated by electrophoresis on 10% SDS‐polyacrylamide gels and transferred onto a PVDF membrane (Millipore). The membranes were blocked with 5% non‐fat dried milk in PBS buffer for 1 h, followed by incubation with primary antibodies overnight at 4°C, and then with secondary antibodies for 1 h at room temperature. Protein blots were finally detected using a chemiluminescence detection kit.

### 4.9 Hemolysis

Hemolysis investigations were conducted by procuring freshly drawn blood from rats into heparinized tubes, followed by subjecting it to centrifugation at a speed of 3000 rpm for a duration of 10 min at a temperature of 4°C. The Red blood cells (RBCs) were washed three times with hemolysis buffer (10 mm Tris, 150 mm NaCl, pH 7.4) and resuspended in the same buffer to a final concentration of 0.25% (v/v). In a 96‐well plate, a volume of 75 µL of the RBC solution was admixed with an equivalent amount of a 2 × **P3** solution, previously dissolved in the hemolysis buffer, to initiate the assay. Negative and positive controls were established using a blank and a buffer containing 1% Triton‐X100, respectively. After incubating the plate for 24 h under gentle agitation, it was centrifuged at 4000 rpm for 10 min at 4°C. Following this, a volume of 100 µL of the supernatant from each well was extracted and transferred to an empty 96‐well plate. The absorbance was then measured at a wavelength of 415 nm utilizing a microplate reader.

### 4.10 Cell Viability Assay

HeLa and NHDF cells were seeded in 96 plates at a density of 5000/well and continued to culture overnight at a 37°C, 5% CO_2_ incubator. Peptide stock solution was prepared in water (containing 1% DMSO) and subsequently diluted to a series of working concentrations. Cells were incubated with peptide work solution for 24 or 48 h. Then the 100 µL 10 %(v/v) CCK8 solution was added to the plate well for 1 h. After incubation, the plates were subjected to the microplate reader to record 450 nm data. The absorbance of the negative controls was subtracted from each sample as a control, and the percent viability was calculated as follows: (Absorbance peptide‐treated cells / Absorbance untreated cells) ×100%. GraphPad Prism 8.0 software was used to fit cytotoxicity curves and calculated IC_50_ values with a non‐linear regression model.

### 4.11 In Vitro Anti‐Tumor Activity Assay Mediated by CDC

HeLa or NHDF cells were seeded in 96‐well plates at a density of 8 × 10^3^ cells per well and cultured for 24 h. Then, the cells were treated with 100 µL of GE11 and GE11‐DNP conjugates at a concentration of 10 µm for 3 h. After washing, the cells were incubated with rabbit anti‐DNP antibody (40 µg/mL) for 1 h, followed by incubation with rabbit complement (1:10 dilution) at 37°C for 10 h. The cell viability was finally measured by the CCK8 kit. The specific cytotoxicity was calculated using the equation CDC (%) = [(A_c_ − A_s_)/(A_c_ − A_max_)] × 100%, where A_c_ is the A_450_ value of cells treated with PBS, A_s_ is the A_450_ value of cells treated with GE11‐DNP conjugates, and A_max_ is the A_450_ value of cells completely lysed with 1% Triton X‐100.

### 4.12 ELISA Protocol for DNP‐Antibody Titer Determination

The mouse DNP‐specific IgE antibody (DNP‐sIgE) quantitative detection kit was purchased from LunChangShuo Biotechnology Co., Ltd. Both the kit and samples were equilibrated at room temperature (25–28°C) for 60 min to ensure thorough temperature stabilization. Standard wells, sample wells, and blank wells were set up. A total of 50 µL of standard at different concentrations was added to the standard wells. 50 µL of test sample (appropriately diluted if necessary) was added to the sample wells, and 50 µL of sample dilution buffer was added to the blank wells. Then, 100 µL of horseradish peroxidase (HRP)‐labeled detection antibody was added to each well. The plate was sealed with a plate sealer and incubated at 37°C in a water bath or incubator for 45 min. After discarding the liquid, the plate was blotted dry on absorbent paper. Each well was filled with 350 µL of wash buffer, allowed to stand for 20 s, and then emptied. The plate was blotted dry again, and this washing process was repeated five times. Subsequently, 50 µL each of substrate A and substrate B was added to every well, followed by incubation at 37°C in the dark for 15 min. Finally, 50 µL of stop solution was added to each well, and the optical density (OD) at 450 nm was measured within 15 min.

### 4.13 In Vivo Antitumor Efficacy

All animal experiments were conducted in accordance with guidelines set forth by the Animal Protection and Ethics Committee of Hunan University and in compliance with the approved protocol by the Institutional Animal Care and Use Committee (IACUC) (Approval Number: HNU‐IACUC‐2021‐102). Female BALB/c nude mice (4 weeks old) were purchased from Gempharmatech Co., Ltd. HeLa cells were suspended in PBS at a density of 8 × 10^7^ cells/mL and injected subcutaneously with 100 µL into the right flank of the nude mice. After tumor implantation for 3 weeks, mice with tumor volumes ranging from 100–120 mm^3^ were grouped and started to receive intraperitoneal administration of 100 µL of anti‐DNP mouse serum every other day. **P3** and **P4** were formulated in PBS and administered subcutaneously at a dose of 5 or 10 mg/kg. The control group received equal volumes of PBS. The tumor sizes of mice were measured, and the corresponding tumor volumes were calculated by the formula *V (*mm^3^ *) =* 0.5 LW^2^ (*L*, length; *W*, width). All mice were sacrificed at the end of the 14‐days treatment cycle. The weights of the tumor were determined after the mice were dissected, followed by the H&E staining analysis of fixed tissue (tumor, heart, liver, spleen, lung, and kidney) with 4% paraformaldehyde.

### 4.14 Statistical Analyses

Data points were collected and compiled in Microsoft Excel. Statistical analyses were performed using GraphPad Prism software. In all cases, significance was defined as * *p* < 0.05, ** *p* < 0.01, *** *p* < 0.001, **** *p* < 0.0001.

## Author Contributions

**Yihui Wang**: conceptualization, methodology, software, data curation, investigation, formal analysis, Writing – original draft. **Xia Wu**: conceptualization, methodology, software, data curation, formal analysis, funding acquisition, writing – review and editing, visualization. **Chenguang Zhao**: investigation, writing – review and editing, validation. **Hong Han**: investigation, supervision, methodology. **Wensha Xie**: investigation, validation, methodology. **Dan Yuan**: funding acquisition, writing – review and editing, resources, validation. **Junfeng Shi**: funding acquisition, writing – original draft, writing – review and editing, resources, project administration, validation.

## Funding

This work was partially supported by the National Natural Science Foundation of China (52573156 to J. Shi, 32401127 to D. Yuan), Natural Science Foundation of Hunan (2024JJ5072 to D. Yuan, 2026JJ50385 to X.W), Natural Science Foundation of Guangdong (2024A1515011057 and 2025A1515012954), Science and Technology and Development Foundation of Shenzhen (JCYJ20230807122008016 and JCYJ20250604190542053 to J. Shi, JCYJ20250604190320027 to X.W).

## Conflicts of Interest

The authors declare no conflicts of interest.

## Supporting information




**Supporting File**: advs78074‐sup‐0001‐SuppMat.docx.

## Data Availability

The data that supports the findings of this study are available in the supplementary material of this article.
