# Supplementary material for: Receptor‐Triggered Peptide Self‐Assembly Enables Multivalent Recruitment of Endogenous Antibodies for Cancer Immunotherapy
Source: Adv Sci (Weinh). 2026 Sep 30:e78074. Online ahead of print. doi: 10.1002/advs.78074 (PMC13626672; doi:10.1002/advs.78074)
Supplement: Supplementary file 1 [file ADVS-9999-e78074-s001.docx]

Supporting Information

Receptor-Triggered Peptide Self-Assembly Enables Multivalent Recruitment of Endogeneous Antibodies for Cancer Immunotherapy

Yihui Wang^1‡^, Xia Wu^2, 3‡*^, Chenguang Zhao^1^, Hong Han^1^, Wensha Xie^1^, Dan Yuan^1*^, Junfeng Shi^1,3*^

^1^ Hunan Provincial Key Laboratory of Animal Models and Molecular Medicine, State Key Laboratory of Chemo/Bio-Sensing and Chemometrics, School of Biomedical Sciences, Hunan University, Changsha, Hunan 410082, China

^2^ Department of Cardiology, the Central Hospital of Xiangtan (Affiliated Hospital of Hunan University) Xiangtan, Hunan 411100, China

^3^ Shenzhen Research Institute of Hunan University, Shenzhen, Guangdong Province 518000, China

E-mail: chemwuxia@hnu.edu.cn, yuandan@hnu.edu.cn, jeff-shi@hnu.edu.cn

Keywords: Self-assembly; EGFR-targeting Peptide; Multivalent effect; Endogenous Antibodies; Cancer immunotherapy

**Table of contents**

**Methods**

**Figure S1.** Synthetic route of **S-1**, **S-2**, and **S-3**.

**Figure S2.** Synthetic route of **DNP-OVA**.

**Figure S3-S21.** Analytical HPLC, ^1^H NMR and MS spectra of designed molecules.

**Figure S22.** Representative western blots of EGFR expression in HeLa, A549, MDA-MB-231 and NHDF cells.

**Figure S23.** Representative 3D Immunofluorescence image of **P3** on HeLa.

**Figure S24.** Serum stability of **P3** and **P4**.

**Figure S25.** TEM image of **P3 and P3 + EGFR** solution.

**Figure S26.** SEM images of **P3** on the surface of HeLa and NHDF cells.

**Figure S27.** The CCK8 assay determinate the cytotoxicity of studied peptides towards

HeLa, A549, MDA-MB-231 and NHDF cells.

**Figure S28.** Hemolysis assays with primary rat blood cells evaluating the hemolytic potential of **P3**.

**Figure S29.** The standard curve of DNP moiety concentration.

**Figure S30.** ELISA results of total anti-DNP titers of pooled antisera collected on mice immunized with the DNP-OVA conjugate.

**Figure S31.** H&E staining of organ sections from HeLa-xenograft mice after peptide

administration.

**Figure S32.** Representative H&E staining images of tumor tissues.

**Peptide Synthesis**

All peptides were synthesized on a CSBio synthesizer using a standard Fmoc solid-phase strategy with 2-chlorotrityl chloride resin, where coupling was mediated by DIEA and HCTU. Following synthesis, the resin-bound peptide was treated with a cleavage cocktail of TFA/phenol/water/triisopropylsilane (88:5:5:2) for 3 hours under a nitrogen atmosphere to effect simultaneous cleavage and side-chain deprotection. After filtration and washing of the resin with excess TFA, the combined filtrates were concentrated, and the crude peptide was precipitated in cold ether. This crude product was then purified by reverse-phase high-performance liquid chromatography (RP-HPLC) and lyophilized to yield a dry powder.

**Synthesis of DNP Derivatives**

**Figure S1.** Synthetic route of **S-1**, **S-2**, and **S-3**.

Compound **2** (3.0 g, 1 eq) was dissolved in DMF, followed by the addition of K_2_CO_3_ (2.57 g, 1.5 eq) and Compound **1** (2.51 g, 1.5 eq), and the reaction was carried out with stirring at 70°C for 15 h. After the reaction, the solution was washed with saturated brine and extracted 3 times with DCM. The combined organic layers were concentrated under reduced pressure and purified by column chromatography (PE/EA, 10:1) to afford 2.3 g of Compound **3** in 37.8% yield.

Compound **3** was dissolved in 50% TFA/DCM and the reaction was stirred at room temperature for 2 h. The DCM was removed by reduced pressure. The crude residue was redissolved in toluene and concentrated under reduced pressure to remove excess TFA, yielding Compound **S-1**, which was used directly without further purification.

Compound **5** (2.0 g, 1 eq) was dissolved in THF, followed by the addition of TEA (1.72 mL, 1.5 eq) and Compound **4** (2.23 g, 1.5 eq), and the reaction was carried out with stirring at 70°C until the reaction was complete monitored by TLC. The reaction solution was washed in saturated saline, extracted with DCM for 3 times. The organic phase was collected and concentrated under reduce pressure and purified by column chromatography (PE/EA, 5:1) to give 2.7 g Compound **6** with 81.0 % yield.

Compound **6** was dissolved in 50% TFA/DCM and the reaction was stirred at room temperature for 2 h. The DCM was removed by reduced pressure. The crude residue was redissolved in toluene and concentrated to remove excess TFA under reduce pressure to give Compound **S-2** which was used directly without purification.

Compound **7** (0.96 g, 1.0 eq) was dissolved in DMF, followed by the addition of K_2_CO_3_ (517 mg, 1.2 eq) and Compound **4** (0.56 g mL, 1.2 eq), and the reaction was carried out with stirring at 70°C for 15 h. The mixture was washed with saturated brine and extracted 3 times with DCM. The organic phases were concentrated under reduced pressure and purified by column chromatography (DCM/MeOH, 30:1) to afford 1.1 g of Compound **8** with 79.8% yield.

Compound **8** was dissolved in 50% TFA/DCM and the reaction was stirred at room temperature for 2 h. The DCM was removed by reduced pressure. After removal of DCM under reduced pressure, the crude residue was redissolved in toluene and concentrated to remove excess TFA, yielding Compound **S-3**, which was used directly without purification.

**Purification and Analysis of GE11**-**DNP Conjugates**

We purified crude peptides using preparative HPLC with an eluent consisting of water (containing 0.1% TFA) and acetonitrile (containing 10% water and 0.1% TFA). All purified peptides were analyzed using analytical HPLC with the same eluent system and LC-MS.

**Synthesis of OVA**-**DNP conjugate**

**Figure S2.** Synthetic route of **DNP-OVA**.

OVA (20 mg) was dissolved in 2 mL of 0.1 M NaHCO₃ solution (pH 9.0) to a concentration of 10 mg/mL. To this solution, 10 mg of DNP-NHS (dissolved in 200 μL DMSO) was added. The reaction mixture was incubated at room temperature for 24 h and then purified using centrifugal filter devices to remove small-molecule reagents and salts. The resulting solution was lyophilized, yielding a yellow powder. Based on a standard curve of 6-((2,4-dinitrophenyl) amino) hexanoic acid measured by spectrophotometry, the DNP loading amount on OVA was determined to be 1.85%.

**Figure S3.** RP-HPLC profile of **S-1**.

**Figure S4.** LC-MS profile of **S-1**.

^1^H NMR (400 MHz, DMSO-d6) δ 8.77 (d, J = 2.8 Hz, 1H), 8.53 (dd, J = 9.2, 2.7 Hz, 1H), 7.62 (d, J = 9.4 Hz, 1H), 4.52 (t, J = 4.9 Hz, 2H), 3.28 (t, J = 5.1 Hz, 2H).

**Figure S5.** ^1^H NMR spectra of compound **S-1**

**Figure S6.** RP-HPLC profile of **S-2**.

**Figure S7.** LC-MS profile of **S-2**.

^1^H NMR (400 MHz, DMSO-d6) δ 8.76 (d, J = 2.9 Hz, 1H), 8.50 (dd, J = 9.4, 2.9 Hz, 1H), 7.62 (d, J = 9.4 Hz, 1H), 4.47 (t, J = 4.4 Hz, 2H), 3.81 (t, J = 4.5 Hz, 2H), 3.64 – 3.56 (m, 6H), 2.96 (t, J = 5.3 Hz, 2H).

**Figure S8.** ^1^H NMR spectra of compound **S-2**

**Figure S9.** RP-HPLC profile of **S-3**.

**Figure S10.** LC-MS profile of **S-3**.

^1^H NMR (400 MHz, DMSO-d6) δ 8.76 (d, J = 2.8 Hz, 1H), 8.50 (dd, J = 9.4, 2.9 Hz, 1H), 7.62 (d, J = 9.4 Hz, 1H), 4.47 (t, J = 4.4 Hz, 2H), 3.79 (t, J = 4.5 Hz, 2H), 3.60 – 3.49 (m, 18H), 2.97 (q, J = 5.4 Hz, 2H).

**Figure S11.** ^1^H NMR spectra of compound **S-3**

**Figure S12.** RP-HPLC profile of **P1**.

**Figure S13.** LC-MS profile of **P1**.

**Figure S14.** RP-HPLC profile of **P2**.

**Figure S15.** LC-MS profile of **P2**.

**Figure S16.** RP-HPLC profile of **P3**.

**Figure S17.** LC-MS profile of **P3**.

**Figure S18.** RP-HPLC profile of **P4**.

**Figure S19.** LC-MS profile of **P4**.

**Figure S20.** RP-HPLC profile of **GE11**.

**Figure S21.** LC-MS profile of **GE11**.

**Figure S22.** Representative western blots of EGFR in (a) HeLa, NHDF, (b) A549 and MDA-MB-231 cells (HEK293T and A431 cells were used as negative and positive controls, respectively).

**(b)**

**Figure S23.** (a) Immunofluorescence map of **P3** on HeLa. (b) 3D Immunofluorescence map of **P3** on HeLa. Blue fluorescence (nucleus), green fluorescence (anti-DNP), Scale bar = 5 μm.

**Figure S24.** Serum stability of **P3** and **P4** over time in the presence of 30% FBS.

**Figure S25.** TEM images of **P3** or **P3** + EGFR protein in (a) BTP buffer (pH = 7.4), (b) PBS (pH = 6.5) and (c) PBS (pH = 8.5) for 24 h. Scale bar: 50 nm.

**Figure S26.** SEM images of 100 µM P3 self-assembled fibrillar network on (a) HeLa and (b) NHDF cell membranes. scale bar = 5 μm/ scale bar = 2 μm.

**Figure S27.** Figure S27. The CCK8 assay determinate the cytotoxicity of studied peptides towards HeLa (a), NHDF (b), MDA-MB-231 (c) and A549 cells(d) for 48 h.

**Figure S28.** Hemolysis assays with rat blood cells evaluating the hemolytic potential of **P3**.

**Figure S29.** The standard curve of DNP moiety concentration as a function of optical density, which was used for determination of DNP loading in OVA-DNP conjugates.

**Figure S30.** ELISA results of total antibody titers of pooled antisera collected on different days from mice immunized with the OVA-DNP conjugate.

**Figure S31.** H&E staining of organ sections, including liver, heart, spleen, lung, and kidney, obtained from mice with EGFR cell xenograft tumors after PBS, 10 mg/kg **P3** and 10 mg/kg **P4** treatment for 14 days. Scale bar = 100 μm.

**Figure S32.** Representative H&E staining images of tumor tissues after treatment with PBS, **P3** and **P4**. Scale bar: 100 μm.
